# Reducing Malaria Transmission through Reactive Indoor Residual Spraying: A Systematic Review

**DOI:** 10.4269/ajtmh.22-0745

**Published:** 2023-12-20

**Authors:** John E. Gimnig, Laura C. Steinhardt, Taiwo Samson Awolola, Daniel Impoinvil, Sarah Zohdy, Kim A. Lindblade

**Affiliations:** ^1^ Entomology Branch, Division of Parasitic Diseases and Malaria, Centers for Disease Control and Prevention, Atlanta, Georgia;; ^2^ Malaria Branch, Division of Parasitic Diseases and Malaria, Centers for Disease Control and Prevention, Atlanta, Georgia;; ^3^ U.S. President’s Malaria Initiative, Entomology Branch, Division of Parasitic Diseases and Malaria, Centers for Disease Control and Prevention, Atlanta, Georgia;; ^4^ Global Malaria Programme, World Health Organization, Geneva, Switzerland

## Abstract

In the final stages of malaria elimination, interventions to reduce malaria transmission are often centered around a confirmed case of malaria, as cases tend to cluster together at very low levels of transmission. The WHO commissioned a systematic review of the literature and synthesis of evidence for reactive indoor residual spraying (IRS) to develop official recommendations for countries. Several electronic databases were searched in November 2020. A total of 455 records were identified and screened; 20 full-text articles were assessed for eligibility. Two cluster-randomized trials met the inclusion criteria for epidemiological outcomes. Risk of bias was assessed using standard criteria. Because one study was a superiority trial in which the comparator included reactive case detection or mass drug administration and the other was a noninferiority trial in which the comparator was proactive, focal IRS, results could not be pooled. In the superiority trial, reactive IRS reduced malaria prevalence by 68% (risk ratio [RR]: 0.32; 95% CI: 0.13–0.80; certainty of evidence: HIGH) compared with no reactive IRS. No difference was observed for clinical malaria (RR: 0.65; 95% CI: 0.38–1.11; certainty of evidence: MODERATE). In the noninferiority study, the mean difference in incidence between reactive IRS and proactive IRS was 0.10 additional case per 1,000 person-years, which was within the prespecified noninferiority bound (95% CI: −0.38 to 0.58; certainty of evidence: MODERATE). The evidence indicates that reactive IRS may be a cost-effective tool for the prevention of malaria in elimination settings. As only two cluster-randomized controlled trials from sub-Saharan Africa were found, additional high-quality studies should be encouraged.

## INTRODUCTION

Indoor residual spraying (IRS) aims to reduce malaria transmission through the application of a residual insecticide to indoor surfaces on which adult female anopheline mosquitoes are likely to rest after ingesting a blood meal. The insecticide reduces the number and longevity of malaria vectors, thereby decreasing transmission of *Plasmodium* species and reducing morbidity and mortality from malaria. Although high-quality cluster-randomized epidemiological studies of the efficacy of IRS are rare,^1^ IRS has been widely credited with the reduction and, in some settings, elimination of malaria, and reviews that include nonrandomized studies have documented substantial reductions in malaria incidence and prevalence.^2^^,^^3^ Most IRS programs in areas of higher transmission are implemented proactively through planned campaigns in which the aim is to spray all houses and other structures in geographic areas. Although logistical challenges and refusals during implementation preclude coverage of every structure, the WHO recommends that IRS programs cover at least 85% of structures in a targeted area. In these settings, IRS is conducted annually or biannually on a large scale (e.g., at a district or provincial level) and is timed to coincide with seasonal increases in mosquito density. In areas with lower transmission or widely dispersed populations, IRS may be implemented more focally by targeting smaller localities with a higher malaria case burden, higher population densities, or greater ease of access. The defining characteristics of proactive IRS interventions, whether implemented in small or large areas, is coverage of all structures in a defined geographic area at a time that coincides with the buildup of vector populations just prior to the onset of peak transmission.

When countries approach malaria elimination, there is a need to adjust strategies to optimize interventions and maximize efficiency. In very low–transmission areas, malaria cases tend to cluster, and many countries pursuing malaria elimination have implemented interventions, including IRS, around parasitologically confirmed malaria cases in an effort to further reduce or interrupt transmission.^4^^,^^5^ Referred to as “reactive” strategies because they are initiated in response to confirmed cases, these actions are dependent on a surveillance system that undertakes case investigations to determine the likely location or source of infection. Reactive IRS targets the residence of, and nearest neighbors to, an index case.^6^^,^^7^ Reactive IRS differs from proactive IRS programs in several ways: First, reactive IRS is implemented in response to individual cases of malaria at the time they are detected rather than based on preexisting knowledge of malaria transmission, whereas proactive IRS is conducted at a prespecified point, such as when there is an expected seasonal increase in mosquitoes. Second, reactive IRS is limited to the household of the index case and the nearest neighbors, whereas proactive IRS is conducted in a prespecified geographic region that usually encompasses a much larger area and covers many more households.

Although proactive IRS has been shown to be effective and is credited with contributing to malaria elimination in many settings, it is an expensive intervention and has not been deployed as widely as insecticide-treated nets. Even with the scale-up of malaria control efforts beginning in 2000, overall IRS coverage across sub-Saharan Africa never rose above 11%.^8^ As resistance to pyrethroids spread, the cost of IRS increased owing to the switch to newer, more effective, but more expensive insecticides and IRS coverage subsequently declined as countries dropped IRS programs that consumed much of their operational budgets.^8^^,^^9^ Given the constraints on funding for malaria prevention and control, cost-effective approaches that maximize impact while minimizing costs are needed. Because reactive IRS is implemented in response to individual cases rather than presumptively spraying large areas, if proven effective, it may present significant cost savings compared with proactive IRS.

Despite potential cost savings and use of reactive IRS by several malaria elimination programs, including China,^10^ reactive IRS was not recommended for elimination settings until June 2022.^11^ This systematic review and synthesis of available evidence and contextual considerations was the basis for the WHO’s recommendations on the use of reactive IRS for the final phase of elimination and prevention of re-establishment.

## MATERIALS AND METHODS

The methods for this systematic review have been described in detail in a previous paper.^12^ An overview of the methods is provided below.

### Population, intervention, comparison, and outcome.

The population included all people living in areas of ongoing or potential malaria transmission. Areas of potential transmission were defined as localities where malaria had been eliminated but efforts continued to prevent re-establishment of transmission. Reactive IRS was defined as IRS in the house of a person with a parasitologically confirmed case of malaria (index case) and a predefined number or radius of neighboring houses.^6^^,^^7^ The type of residual insecticide sprayed for reactive IRS included all those that are prequalified by the WHO plus dichlorodiphenyl trichloroethane.^13^ The primary comparison was considered no reactive IRS, though comparison of reactive IRS to proactive IRS was also considered. The coverage of any co‐interventions such as insecticide-treated nets, larval source management, topical repellents, spatial repellents, mass drug administration, or case management had to be balanced between intervention and comparison arms or periods. Critical transmission-related outcomes were measured at the population level and included the incidence and prevalence of malaria. In addition, potential negative outcomes such as adverse events and development of insecticide resistance were included if available.

### Search strategy.

Eight databases (Medline, Embase, Global Health, Cochrane Library, CINAHL, Scopus, Clinicaltrials.gov, and Global Index Medicus) were searched using three sets of key terms, which varied slightly for different databases. All searches included the key term “malaria.” Additional key terms specifying vector control included “indoor residual spray” or “IRS” or “vector control” or “focal vector.” Key terms to identify studies related to reactive IRS included “reactive” or “index” or “close contact.” The detailed search strategy is available in Supplemental Table 1.

### Contextual factors.

Evidence for contextual factors such as feasibility, acceptability, health equity, and financial considerations, as defined previously, was summarized when available.

### Data collection and analysis.

The selection of studies, data extraction, and data synthesis have been described previously.^12^ Assessment of risk of bias in individual studies was conducted using the Cochrane Risk of Bias tool for cluster-randomized studies.^14^ The following potential effect modifiers were abstracted to permit subgroup analyses if possible: level of transmission; malaria parasite species of the index case; size or population of intervention area around the index case; and vector species. In addition, characteristics of the malaria vectors and malaria epidemiology in the area were abstracted to provide additional context to the studies.

The quality and strength of the evidence for each outcome was evaluated using the Grading of Recommendations, Assessment, Development and Evaluation (GRADE) approach.^15^

## RESULTS

### Search results.

**Figure 1.**
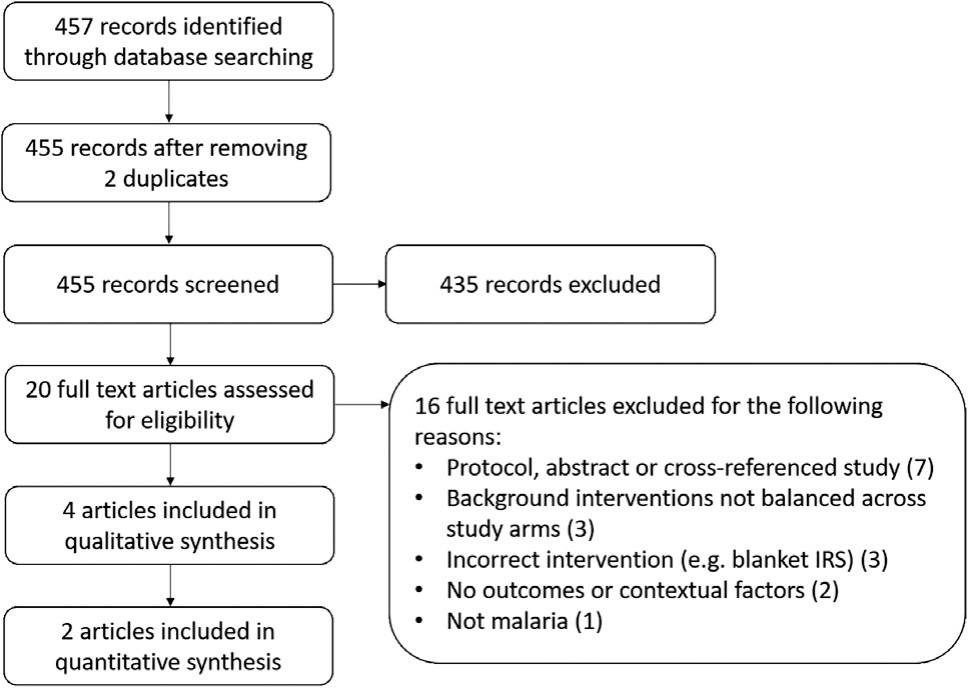
PRISMA flow diagram. IRS = indoor residual spraying; PRISMA = Preferred Reporting Items for Systematic Reviews and Meta-Analyses.

A total of 457 articles were identified from searching electronic databases, registers, and other sources. After de-duplication, 455 articles were screened against title and abstract eligibility, 20 studies were assessed for full-text eligibility criteria, and four studies were included: two cluster-randomized controlled trials (cRCTs) with outcome data (one of which also included contextual information) and two studies reporting only on contextual factors (Figure 1). Full-text studies that did not meet the eligibility criteria are listed with their reasons for exclusion in Supplemental Table 2.

Two eligible studies were identified from the full-text review that provided quantitative data for analysis. One was a cRCT from Namibia, a 2 × 2 superiority trial in which reactive IRS (referred to as “reactive vector control” in the article) was implemented with either reactive case detection or reactive mass drug administration (referred to as “reactive focal mass drug administration” in the paper) and was compared with no reactive IRS.^7^ The second was a cRCT from South Africa, a noninferiority study comparing reactive IRS (called “targeted IRS” in the article) with proactive, focal IRS (referred to as “standard IRS” in the article).^6^ Proactive IRS was considered focal, as only a geographic subset of the targeted districts received IRS. In addition, IRS was conducted in the houses of index cases if the case was determined to be locally acquired and if the house had not already been sprayed. Details of the characteristics of each study, including details on the implementation of reactive IRS, the number of clusters and total population in both treatment and comparison arms, baseline incidence of malaria, and insecticide susceptibility of local vector populations, are provided in Table 1 and in Supplemental Tables 3 and 4.

**Table 1:** Summary characteristics of included studies

Reference	Country (years of intervention)	Study Design	Baseline Transmission	Reactive IRS		Outcomes Reported
				Insecticide (target dose) and Targeted Households	Number of Clusters, Population Targeted, and Coverage	
Hsiang et al.^7^	Namibia (2017)	Cluster-randomized controlled trial, superiority design	35.9 Cases per 1,000 population per year	Pirimiphos-methyl (1 g/m^2^); up to seven households within 500 m of index case	Clusters = 28 Population = 9,464*; coverage of index houses = 81.6%; coverage of targeted houses = 93.3%; overall coverage = approximately one-third of houses	Cumulative incidence beginning 8 weeks after the first index case in each cluster; prevalence of infection in an endline survey; adverse events
Bath et al.^6^	South Africa (2015–2017)	Cluster-randomized controlled trial, noninferiority design	1–5 Cases per 1,000 person-years	Deltamethrin; up to eight households within 200 m of index case	Clusters = 31; population = 204,237; overall coverage in reactive IRS = 5%; overall coverage in proactive, focal IRS = 30%	Mean cluster-level incidence; adverse events (malaria deaths); contextual factors: financial and economic considerations

IRS = indoor residual spraying.

* Estimated from average cluster size of 338.

Both studies measured the incidence of clinical malaria through passive case detection at health facilities with parasitological confirmation by microscopy or rapid diagnostic tests. Adverse events were measured through active monitoring in both studies and additionally through self-report in the Namibia study. Insecticide resistance was not measured in either study as a potential outcome of the intervention, although susceptibility to the insecticides used for IRS was reported for both studies. Overall, data from the two studies could not be pooled for any outcome because of the different study designs, which resulted in different comparisons. Subgroup analyses were not possible given the small number of studies.

### Primary epidemiological outcomes.

**Figure 2.**
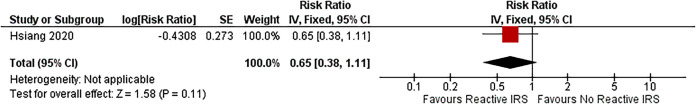
Forest plot of comparison: reactive IRS versus no reactive IRS on the incidence of clinical malaria, superiority design. The authors calculated the effect size using marginal effects post-estimation (to account for reactive drug administration in half the clusters) after a regression model; thus, the 95% CI bounds are not balanced around the point estimate. Because the Review Manager software can only accommodate balanced CIs, the 95% CI contains the correct upper bound but is artificially narrow. IV = inverse variance; IRS = indoor residual spraying; SE = standard error.

**Figure 3.**
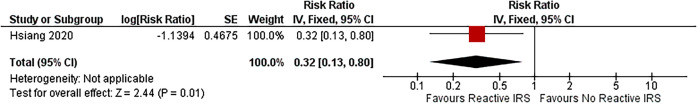
Forest plot of comparison: reactive IRS versus no reactive IRS on the prevalence of malaria, superiority design. The authors calculated the effect size using marginal effects post-estimation (to account for reactive drug administration in half the clusters) after a regression model; thus, the 95% CI bounds are not balanced around the point estimate. Because the Review Manager software can only accommodate balanced CIs, the 95% CI contains the correct upper bound but is artificially narrow. IV = inverse variance; IRS = indoor residual spraying; SE = standard error.

The Namibia study reported a 35% reduction in the cumulative incidence of clinical malaria in clusters randomized to reactive IRS compared with those randomized to no reactive IRS (risk ratio [RR]: 0.65; 95% CI: 0.38–1.11 Figure 2).^7^ Although results for several models were reported in the main paper, this review used the model that was specified as the primary analysis in the study protocol and adjusted only for baseline incidence; results from additional models that adjusted for other covariates are included in Supplemental Figure 1.^16^ When adjusting only for baseline incidence, the Namibia study reported that reactive IRS reduced the prevalence of malaria as measured by quantitative polymerase chain reaction by 68% (RR: 0.32; 95% CI: 0.13–0.80) when results from the arm with reactive IRS plus the reactive case detection arm were compared with the arm with reactive case detection only (Figure 3).^7^

**Figure 4.**
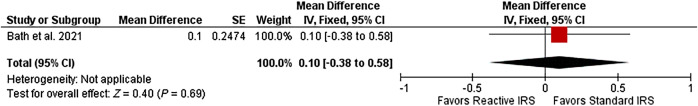
Forest plot of comparison: reactive IRS versus proactive, focal IRS on the incidence of clinical malaria, noninferiority design. IV = inverse variance; IRS = indoor residual spraying; SE = standard error.

The South Africa study prespecified the noninferiority bound as one case per 1,000 person-years. The mean difference in incidence was estimated as 0.10 additional case per 1,000 person-years in the reactive IRS arm (95% CI: −0.38 to 0.58; Figure 4), indicating that reactive IRS was noninferior to proactive, focal IRS. When adjusted for province, the results did not change substantially (mean difference: 0.14; 95%CI: −0.27 to 0.55). Additional analyses by year and province are presented in Supplemental Figures 2 and 3.

The study in Namibia reported adverse events (headache, dizziness, diarrhea, and others) and found that more participants reported adverse events in the study arms that did not receive reactive IRS (*N* = 12, 0.27%) than those that did (*N* = 6, 0.13%). None of the adverse events were considered to be related to IRS.^7^ In the South African study, nine deaths due to malaria were reported in the reactive IRS arm out of a population of 204,237, whereas in the proactive, focal IRS arm, a total of 12 malaria deaths were reported out of a population of 189,150.^6^

#### Risk of bias.

In both studies, the risk was determined to be low for each of the following potential sources of bias: the randomization process; identification or recruitment of participants into clusters; missing outcome data; and measurement of the outcome (Supplemental Figure 4). With respect to bias due to deviations from intended intervention, no deviations were reported in the Namibia study.^7^ However, the average number of households sprayed around each index case in the South Africa study was 3.7, well below the target of eight households per index case. The low coverage could have resulted in an underestimate of the efficacy of reactive IRS; therefore, we judged there to be a high risk of bias for this study.^6^ Although the study from Namibia reported results in the main paper that were not consistent with the prespecified analysis plan, the results of the planned analysis were reported in the supplementary material and used in the data synthesis. As a result, both studies were reported to have a low risk of bias in selection of the reported result. The GRADE approach indicated that the quality of evidence for the comparison of reactive IRS with no reactive IRS was moderate to high (Table 2). For the comparison of reactive IRS to targeted proactive IRS, the certainty of evidence was downgraded due to the indirectness of the comparison and was considered moderate (Table 3). It was not possible to estimate potential publication bias.

**Table 2:** Reactive IRS compared with reactive case detection or reactive mass drug administration (superiority design)

Outcomes	Anticipated Absolute Effects		Relative Effect (95% CI)	Number of Trials, Participants	Certainty of the Evidence (GRADE)	Comments
	Risk with Reactive IRS (95% CI)	Risk with Standard of Care (95% CI)				
Incidence of clinical malaria	30.2 cases/ 1,000 PY (15.0–45.5)	38.9 cases/ 1,000 PY (20.7–57.1)	RR = 0.65 (0.19–1.11)	One trial; 56 clusters (55 analyzed: 28 reactive IRS, 27 no reactive IRS); 18,303 participants (9,464 reactive IRS, 9,352 no reactive IRS)	MODERATE owing to imprecision	There is probably no difference in the incidence of clinical malaria in clusters with reactive IRS plus RACD or rMDA compared with clusters with RACD or rMDA; imprecision resulted from wide confidence intervals
Parasite prevalence	2.92% (2.13–3.99)	4.07% (2.92–5.64)	PR = 0.32 (0.15–0.80)	One trial; 56 clusters (55 analyzed: 28 reactive IRS, 27 no reactive IRS); 4,082 participants in endline survey (2,052 reactive IRS, 2,030 no reactive IRS)	HIGH	The prevalence of malaria in clusters with reactive IRS plus RACD or rMDA was lower than in clusters with RACD or rMDA

GRADE = Grading of Recommendations, Assessment, Development and Evaluation; IRS = indoor residual spraying; rMDA = reactive mass drug administration; PR = prevalence ratio; PY = person-year; RACD = reactive case detection; RR = risk ratio.

**Table 3:** Reactive IRS compared with proactive IRS (noninferiority design)

Outcomes	Anticipated Absolute Effects		Relative Effect (95% CI)	Number of Trials, Participants	Certainty of the Evidence (GRADE)	Comments
	Risk with Reactive IRS (95% CI)	Risk with Standard of Care (95% CI)				
Incidence of clinical malaria	1.05 cases/1,000 PY (0.72–1.38)	0.95 case/1,000 PY (0.58–1.32)	MD = 0.10 (−0.38 to 0.59)	One trial; 62 clusters (31 reactive IRS, 31 proactive, focal IRS); 393,387 participants (204,237 reactive IRS, 189,150 proactive, focal IRS)	MODERATE owing to indirectness	There is probably no difference in the incidence of clinical malaria in clusters with reactive IRS compared with clusters with focal proactive IRS; indirectness due to comparison with proactive, focal IRS

GRADE = Grading of Recommendations, Assessment, Development and Evaluation; IRS = indoor residual spraying; MD = mean difference; PY = person-years.

### Contextual factors.

Three studies (one of the cRCTs plus two other articles) reported on contextual factors related to acceptability, feasibility, and financial and economic considerations.^6^^,^^10^^,^^17^ A study in China examined the feasibility of reactive IRS in the context of the 1-3-7 elimination strategy, which aimed to report cases within 1 day of diagnosis, investigate cases within 3 days, and respond within 7 days.^10^ The response included reactive IRS, and the aim of the study was to assess whether targets were met. All (100%) malaria cases were reported within the first 24 hours; 98% of case investigations were done within 3 days; and 96% of foci investigations were completed in 7 days. Among active foci, 96% were offered treatment, reactive case detection, and reactive IRS according to the planned schedule, demonstrating the feasibility of implementing reactive IRS.

A study in Namibia conducted alongside the efficacy trial assessed the community acceptance of reactive IRS.^17^ This mixed-methods study included measurement of refusal rates, key informant interviews, focus group discussions, and an endline cross-sectional survey that measured satisfaction with reactive IRS. In the year prior to full implementation of the trial, the refusal rate for reactive IRS was high (13.9%). Refusals were due to lack of notification before arrival and reluctance to move furniture at short notice. In the second year, advance notification was provided and <1% of households refused IRS. In key informant interviews and focus group discussions, respondents generally considered reactive IRS to be a useful tool for malaria prevention, and participants in study arms that did and did not receive reactive IRS indicated a desire to have their houses sprayed. In an endline survey, 616 of 624 respondents (98.7%) from the reactive IRS clusters indicated they would participate in the same intervention again.

Lastly, the cRCT conducted in South Africa also included estimates of the cost-effectiveness of reactive IRS in comparison to that of proactive, focal IRS.^6^ Over the 2-year study, the average annual economic cost was $184,319 per 100,000 population in the proactive, focal IRS arm compared with $88,258 per 100,000 population in the reactive IRS arm, a 52% cost savings. Overall, reactive IRS saved $7,845 (95% CI: $2,902–$64,907) per additional disability-adjusted life-years incurred. At the incidence observed in the trial, switching from proactive, focal IRS to reactive IRS would have a 94–98% probability of being the cost-effective choice for South Africa. It was estimated that reactive IRS would remain the preferred strategy up to an incidence of 2.0 to 2.7 cases per 1,000 person-years using the cost-effectiveness thresholds established for South Africa.

## DISCUSSION

Although many countries approaching elimination or working to prevent re-establishment are implementing reactive IRS, published data on the impact of these efforts are lacking (K. A. Lindblade, personal communication), and the WHO did not recommend reactive IRS as an intervention to reduce malaria transmission in elimination settings until June 2022. Two cRCTs of reactive IRS from countries in sub-Saharan Africa were identified and included in this systematic review. During the period covered by these studies, South Africa, but not Namibia, was considered to be approaching malaria elimination based on an arbitrary cutoff of reporting fewer than 10,000 malaria cases annually.^6^^,^^7^

Although the two studies differed in design and other characteristics, both suggest a potential impact of reactive IRS. The Namibia study showed a significant effect on parasite prevalence. The impact of reactive IRS on the incidence of clinical malaria in Namibia was not statistically significant. However, when models were adjusted for response time and coverage or co-interventions, reactive IRS was shown to have a significant effect on malaria incidence, suggesting that operational considerations could affect outcomes. In South Africa, reactive IRS was considered noninferior to proactive, focal IRS, which is recommended by the WHO and has been shown to be effective in reducing the transmission of malaria, particularly in areas of low, unstable transmission.^1^

The frequency of adverse events was low in both studies. In the South Africa study, only malaria-related deaths were evaluated as potential adverse events to ensure that reactive IRS did not result in an increased mortality rate owing to lower efficacy; however, malaria-related mortality was lower in the reactive IRS arm than in the proactive, focal IRS arm. In Namibia, the study team conducted both passive and active follow-up for adverse events but reported only 23 adverse events among 18 individuals, of whom 6 were in the reactive IRS arm and 12 were in study arms without reactive IRS; none of the adverse events were considered related to the IRS. Neither study was designed to monitor for changes in insecticide resistance among the local vector populations.

This review found that reactive IRS reduces malaria prevalence, probably reduces the incidence of clinical malaria, and probably results in little to no difference in adverse events compared with no reactive IRS. This review suggests that in comparison to proactive, focal IRS, reactive IRS probably results in little to no difference in the incidence of clinical malaria, suggesting that it is as equally effective as proactive, focal IRS. In addition, the study in South Africa compared the cost-effectiveness of reactive IRS to that of proactive, focal IRS and determined that the former is likely to be more cost-effective than the latter in areas where the incidence of clinical malaria is no more than 2.0 to 2.7 cases per 1,000 person-years. As reactive interventions depend upon the implementation of case investigations, which are likely not possible until the incidence is well below 1 per 1,000 population, this finding suggests that reactive IRS is likely to be more cost-effective than proactive, focal IRS in areas where case investigations can be implemented.

There were some important differences in the design and implementation of reactive IRS in the two studies. The Namibia study was a superiority trial in which reactive IRS was compared with no reactive IRS, whereas the South Africa study was a noninferiority study comparing reactive IRS with proactive, focal IRS, which had been implemented in the area for years.^6^^,^^7^ The insecticide used in Namibia was pirimiphos-methyl (an organophosphate), whereas the South Africa study used deltamethrin (a pyrethroid), although it is unlikely that the selection of insecticide affected the outcomes. Susceptibility to deltamethrin was reported in the trial sites in South Africa, and bioassays conducted in Namibia indicated full susceptibility of *Anopheles gambiae* complex mosquitoes to pirimiphos-methyl. These insecticides have varying durations of efficacy^18^; however, given the short duration of the transmission in each site, this is also unlikely to have substantially affected the results in either site. The implementation of reactive IRS in Namibia targeted a minimum of seven houses in a 500-m radius around the index case in an attempt to achieve 80% coverage of all houses, whereas in South Africa, up to eight houses were targeted in a 200-m radius around the index case. The overall coverage of houses in the study area that were sprayed by the reactive IRS strategy differed considerably between studies, with one-third of households estimated to have been sprayed in Namibia compared with 5% in South Africa. This difference is likely a result of the significantly lower level of malaria transmission in South Africa but could also have arisen from different sizes of the radii and population density.

Although the certainty of evidence was moderate to high, only two studies were included in this review. Many countries are now nearing elimination and are either considering or currently implementing reactive strategies such as reactive IRS. Additional studies would further strengthen the evidence base for reactive IRS as well as better define the specific conditions under which it might be more feasible and cost-effective compared with proactive IRS.

## Supplemental Materials

10.4269/ajtmh.22-0745 Supplemental Materials

## References

[1] Pluess B Tanser FC Lengeler C Sharp BL, 2010. Indoor residual spraying for preventing malaria. Cochrane Database Syst Rev 4: CD006657. 10.1002/14651858.CD006657.pub2 PMC6532743 20393950

[2] Kim D Fedak K Kramer R, 2012. Reduction of malaria prevalence by indoor residual spraying: a meta-regression analysis. Am J Trop Med Hyg 87: 117–124. 22764301 10.4269/ajtmh.2012.11-0620 PMC3391035

[3] Zhou Y , 2022. Effectiveness of indoor residual spraying on malaria control: a systematic review and meta-analysis. Infect Dis Poverty 11: 83. 35870946 10.1186/s40249-022-01005-8 PMC9308352

[4] Cotter C Sturrock HWJ Hsiang MS Liu J Phillips AA Hwang J Smith Gueye C Fullman N Gosling RD Feachem RGA, 2013. The changing epidemiology of malaria elimination: new strategies for new challenges. Lancet 382: 900–911. 23594387 10.1016/S0140-6736(13)60310-4 PMC10583787

[5] Stresman G Whittaker C Slater HC Bousema T Cook J, 2020. Quantifying *Plasmodium falciparum* infections clustering within households to inform household-based intervention strategies for malaria control programs: an observational study and meta-analysis from 41 malaria-endemic countries. PLoS Med 17: e1003370. 33119589 10.1371/journal.pmed.1003370 PMC7595326

[6] Bath D , 2021. Effectiveness and cost-effectiveness of reactive, targeted indoor residual spraying for malaria control in low-transmission settings: a cluster-randomised, non-inferiority trial in South Africa. Lancet 397: 816–827. 33640068 10.1016/S0140-6736(21)00251-8 PMC7910276

[7] Hsiang MS , 2020. Effectiveness of reactive focal mass drug administration and reactive focal vector control to reduce malaria transmission in the low malaria-endemic setting of Namibia: a cluster-randomised controlled, open-label, two-by-two factorial design trial. Lancet 395: 1361–1373. 32334702 10.1016/S0140-6736(20)30470-0 PMC7184675

[8] WHO , 2022. World Malaria Report. Geneva, Switzerland: World Health Organization.

[9] Oxborough RM, 2016. Trends in US President’s Malaria Initiative-funded indoor residual spray coverage and insecticide choice in sub-Saharan Africa (2008–2015): urgent need for affordable, long-lasting insecticides. Malar J 15: 146. 26957210 10.1186/s12936-016-1201-1 PMC4784374

[10] Zhou SS , 2015. China’s 1-3-7 surveillance and response strategy for malaria elimination: is case reporting, investigation and foci response happening according to plan? Infect Dis Poverty 4: 55. 26654106 10.1186/s40249-015-0089-2 PMC4674909

[11] WHO , 2022. Guidelines for Malaria Vector Control (WHO/UCN/GMP/2022.01 Rev. 2). Geneva, Switzerland: World Health Organization. 30844152

[12] Tusell M , 2024. Development of systematic reviews to inform WHO’s recommendations for elimination and prevention of re-establishment of malaria: Methodology. Am J Trop Med Hyg 110 * (* *Suppl 4* *): * 11–16. 38118164 10.4269/ajtmh.22-0740 PMC10993789

[13] WHO , 2022. *Vector Control Product List*. Available at: https://extranet.who.int/pqweb/vector-control-products/prequalified-product-list. Accessed July 5, 2022.

[14] Eldridge S Campbell M Campbell M Drahota A Giraudeau B Reeves B Siegfried N Higgins J, 2021. *Revised Cochrane Risk of Bias Tool for Randomized Trials (RoB 2): Additional Considerations for Cluster-Randomized Trials (RoB 2 CRT)*. Available at: https://www.riskofbias.info/welcome/rob-2-0-tool/rob-2-for-cluster-randomized-trials. Accessed December 4, 2023.

[15] Atkins D , 2004. Grading quality of evidence and strength of recommendations. BMJ 328: 1490. 15205295 10.1136/bmj.328.7454.1490 PMC428525

[16] Medzihradsky OF , 2018. Study protocol for a cluster randomised controlled factorial design trial to assess the effectiveness and feasibility of reactive focal mass drug administration and vector control to reduce malaria transmission in the low endemic setting of Namibia. BMJ Open 8: e019294. 10.1136/bmjopen-2017-019294 PMC5829876 29374672

[17] Roberts KW , 2021. Community acceptance of reactive focal mass drug administration and reactive focal vector control using indoor residual spraying, a mixed-methods study in Zambezi region, Namibia. Malar J 20: 162. 33752673 10.1186/s12936-021-03679-1 PMC7986500

[18] Dengela D Seyoum A Lucas B Johns B George K Belemvire A Caranci A Norris LC Fornadel CM, 2018. Multi-country assessment of residual bio-efficacy of insecticides used for indoor residual spraying in malaria control on different surface types: results from program monitoring in 17 PMI/USAID-supported IRS countries. Parasit Vectors 11: 71. 29382388 10.1186/s13071-017-2608-4 PMC5791726

