# Supplementary material for: Reducing Malaria Transmission through Reactive Indoor Residual Spraying: A Systematic Review
Source: Am J Trop Med Hyg. 2023 Dec 20;110(4 Suppl):94–100. doi: 10.4269/ajtmh.22-0745 (PMC10993783; doi:10.4269/ajtmh.22-0745)
Supplement: Supplemental Materials [file tpmd220745.SD1.pdf]

Supplemental Data

Reducing Malaria Transmission through Reactive Indoor Residual Spraying: a systematic review

John E. Gimnig, Laura Steinhardt, Taiwo Samson Awolola, Daniel Impoinvil, Sarah Zohdy and

Kim A. Lindblade

Supplemental Table 1. Search strategy

Database	Strategy	Records
**Medline (OVID) 1946-**	Malaria*<br>AND<br>(indoor* ADJ5 residual ADJ5 spray*) OR (in-door* ADJ5 residual ADJ5 spray*) OR pirimiphos-methyl OR vector control OR focal vector OR IRS<br>AND<br>Reactive OR index OR close contact*	147
**Embase (OVID) 1988-**	Malaria*<br>AND<br>(indoor* ADJ5 residual ADJ5 spray*) OR (in-door* ADJ5 residual ADJ5 spray*) OR pirimiphos-methyl OR vector control OR focal vector OR IRS<br>AND<br>Reactive OR index OR close contact*<br>NOT pubmed/medline	230<br><br>-147 duplicates<br><br>=83 unique items
**Global Health (OVID) 1910_**	Malaria*<br>AND<br>(indoor* ADJ5 residual ADJ5 spray*) OR (in-door* ADJ5 residual ADJ5 spray*) OR pirimiphos-methyl OR vector control OR focal vector OR IRS<br>AND<br>Reactive OR index OR close contact*	214<br><br>-122 duplicates<br><br>= 92 unique items
**Cochrane Library**	Malaria*:ti,ab<br>AND	22<br><br>-9 duplicates

	((indoor* NEAR/5 residual NEAR/5 spray*) OR (in-door* NEAR/5 residual NEAR/5 spray*) OR pirimiphos-methyl OR "vector control" OR "focal vector" OR IRS):ti,ab<br>AND<br>(Reactive OR index OR "close contact*"):ti,ab.	=13 unique items
**CINAHL (EbscoHost)**	Malaria*<br>AND<br>(indoor* N5 residual N5 spray*) OR (in-door* N5 residual N5 spray*) OR pirimiphos-methyl OR "vector control" OR "focal vector" OR IRS<br>AND<br>Reactive OR index OR "close contact*"	20<br>-18 duplicates<br>=2 unique items
**Scopus**	TITLE-ABS-KEY(Malaria*) AND TITLE-ABS-KEY((indoor W/5 residual W/5 spray*) OR (in-door* W/5 residual W/5 spray*) OR pirimiphos-methyl OR "vector control" OR "focal vector" OR IRS) AND TITLE-ABS-KEY(Reactive OR index OR "close contact*")	246<br>-177 duplicates<br>=69 unique items
**Clinicaltrials.gov**	Malaria | indoor residual spraying | completed	12<br>-2 duplicates<br>=10 unique items
**Global Index Medicus**	Malaria*<br>AND<br>"indoor residual spraying" OR "in-door residual spraying" OR pirimiphos-methyl OR "focal vector"	42<br>-2 duplicates<br>=40 unique items

Supplemental Table 2. List of studies excluded after full review and primary reasons for exclusion

Study	Reference	Primary reason for exclusion
Chadee 1992	1	Background interventions not balanced across study arms
Chanda 2018	2	Background interventions not balanced across study arms
Galappaththy 2012	3	No outcomes or contextual factors
Galatas 2020	4	Incorrect intervention
Gerardin 2017	5	Incorrect intervention~~Protocol, abstract or cross-referenced study~~
Gueye 2018	6	Protocol, abstract or cross-referenced study
Hetzel 2020	7	No outcomes or contextual factors
Huda 2019	8	Not malaria
Kandeel 2016	9	Incorrect intervention
Karunasena 2019	10	Background interventions not balanced across study arms
Kleinschmidt 2017	11	Protocol, abstract or cross-referenced study
Larson 2015	12	Incorrect intervention
London School of Hygiene and Tropical Medicine 2015	13	Protocol, abstract or cross-referenced study
Medzihrandsky 2018	14	Protocol, abstract or cross-referenced study
Ntuku 2017	15	Protocol, abstract or cross-referenced study
University of California 2016	16	Protocol, abstract or cross-referenced study

Supplemental Table 3. Detailed characteristics of the Namibia study[17]

Study characteristics	
METHODS	
**Study dates**	1 January 2017 to 31 December 2017
**Location(s) of study:**	Zambezi region of northern Namibia
**Baseline malaria endemicity**	• Malaria incidence: 23.5 cases per 1000 per year in 2013 and 2014 but an outbreak in 2016 resulted in 35.9 per 1000 per year<br>• Malaria prevalence: 2.2% prevalence by loop-mediated isothermal amplification in 2015
**Peak transmission season**	January to June
**Malaria species**	*P. falciparum*
**Vector species**	*Anopheles arabiensis*
**Entomologic inoculation rate (EIR)**	Not described
**Insecticide resistance context**	• 100% susceptibility to pirimiphos-methyl<br>• 98% susceptibility to DDT<br>• 71% susceptibility to deltamethrin<br>• No mutations observed in the voltage gated sodium channel
**Study design**	Cluster randomized controlled study, superiority design
**Statistical power calculation**	Incidence: The study had 80% power to detect a 50% or greater relative reduction in incidence in clusters receiving either reactive focal mass drug administration (rfMDA) or reactive IRS alone, and to detect a 75% relative reduction in incidence in clusters receiving combined interventions, with 14 clusters per study arm (harmonic mean of 276 individuals per cluster), based on an anticipated baseline annual incidence of 24·4 cases per 1000 individuals for the reactive case detection only arm, a coefficient of variation of 0·95 based on previous incidence (in 2013 and 2014), and a two-sided significance level of 0·05.<br><br>Prevalence: For the cross-sectional survey, 25 households in each cluster were sampled. Assuming a mean household size of four individuals and that 20% of households would not respond to the survey, a sample size of 5040 individuals provided 80% power to detect a 55% relative reduction in prevalence in individuals receiving either rfMDA or reactive IRS alone, and to detect an 83% relative reduction in prevalence in those receiving the combined interventions, assuming 5% prevalence of infection detected by qPCR in the reactive case detection only arm, a coefficient of variation of 1·0, and a two-sided significance level of 0·05.
**Clusters or groups**	Unit of non-randomized group allocation: Enumeration areas<br>Number of clusters selected: 56 (28 reactive IRS, 28 no reactive IRS)<br>Analyzed: 55 (one cluster allocated to no reactive IRS was not included in the analysis as no index cases were observed)<br>Average cluster size: 4621

	Design features of the clusters: Enumeration areas were eligible for inclusion if they were located within the catchment area of one of the 11 study health-care facilities. Enumeration areas that had no reported incident cases or incomplete incidence data from 2012–14 were excluded
PARTICIPANTS	
**Population targeted**	Total: 18,303<br>Intervention: 9,464 (estimated from mean cluster size of 338)<br>Comparator: 9,352 (estimated from mean cluster size of 334)
**Eligibility**	All persons living in eligible clusters
INTERVENTION	
**Insecticide and dose**	Pirimiphos-methyl, $1g/m^2$
**Targeted coverage around index case**	80% of households within 500m of the index case with a target of at least 7 households around each index case; households that had been previously sprayed due to overlap with another index case were not eligible to be sprayed again
**Actual IRS coverage**	Index case: 81.6%<br>Targeted households around index case: 93.3%<br>Overall: approximately 1/3 of households in reactive IRS clusters were sprayed
**Background/co-interventions**	RACD and rfMDA were conducted in half of the clusters. Each co-intervention was balanced between reactive IRS and no reactive IRS arms
COMPARISON	
**Treatment arms**	Reactive IRS, with either RACD or rfMDA, was compared to no reactive IRS, with either RACD or rMDA in a superiority trial
OUTCOMES	
**Incidence of clinical malaria**	Measurement: Monthly incidence measured by microscopy at village hospitals; diagnosis by RDT or microscopy
**Prevalence of malaria infection**	Measurement: Cross-sectional mass blood survey at end of the study; diagnosed by qPCR<br>Sample size: 2,052 (reactive IRS); 2,030 (no reactive IRS)
**Adverse effects**	Participants were instructed to report adverse events to the on-call study nurse or the nearest health facility.

Supplemental Table 4. Detailed characteristics of the South Africa study[18]

**Study characteristics**	
METHODS	
**Study dates**	1 August 2015 to 31 July 2017
**Location(s) of study:**	Limpopo and Mpumalanga Provinces of South Africa
**Baseline malaria endemicity**	Mean annual malaria case incidence per 1000 population from 2010-2015 was 1.05 in proactive, focal IRS clusters and 0.88 in reactive IRS clusters.
**Peak transmission season**	January to June
**Malaria species**	*P. falciparum*
**Vector species**	*Anopheles arabiensis*
**Entomologic inoculation rate (EIR)**	Not described
**Insecticide resistance context**	Resistance to DDT or pyrethroids has not previously been observed in the study area
**Study design**	Cluster randomized controlled study, non-inferiority design
**Statistical power calculation**	Mean incidence was assumed to be 2.2 locally acquired infections per 1000 person-years. Assuming a coefficient of variation of 0.5, the trial required 31 clusters per arm with ~6000 people for 2 years (i.e. 12,000 person years per cluster) to show non-inferiority within a margin of 1 case per 1000 person-years.
**Clusters or groups**	Unit of non-randomized group allocation: Census wards<br>Number of clusters selected: 62 (31 reactive IRS, 31 proactive, focal IRS)<br>Analyzed: 62<br>Average cluster size: 6,588 (reactive IRS); 6,102 (proactive, focal IRS)<br>Design features of the clusters: Clusters had to have a history of local cases in at least one year in the 5 years prior to the study. Where possible, clusters were separated by natural boundaries or uninhabited areas
PARTICIPANTS	
**Population targeted**	Total: 393,387<br>Reactive IRS: 204,237<br>Proactive, focal IRS: 189,150
**Eligibility**	All persons living in eligible clusters
INTERVENTION	
**Insecticide and dose**	Deltamethrin, 20mg/m^2
**Targeted coverage around index case**	80% of households within 500m of the index case with a target of at least 7 households around each index case; households that had been previously sprayed due to overlap with another index case were not eligible to be sprayed again

**Actual IRS coverage**	Proactive, focal IRS: 30%<br>Reactive IRS: 5%
**Background/co-interventions**	Reactive case detection was done in all clusters; reactive case detection involved investigation of index cases and testing and referral of household members
COMPARISON	
**Treatment arms**	Reactive IRS was compared to proactive focal IRS. Proactive focal IRS targeted at-risk areas which were identified based on the proximity to rivers and streams, the number of malaria cases in the previous season, and malaria control programme expert opinion. The proactive IRS did include some reactive IRS where index households of malaria cases were sprayed if they had not been sprayed previously. IRS began in August and was concluded in December each year.
OUTCOMES	
**Incidence of clinical malaria**	<u>Measurement</u>: Monthly incidence measured by microscopy at village hospitals; diagnosis by RDT or microscopy
**Adverse effects**	Malaria deaths were detected through the routine health system.

Supplemental Figure 1. Plot of various models of the effect of reactive IRS versus no reactive IRS from the Namibia study[17]

Study or Subgroup	log[Risk Ratio]	SE	Risk Ratio IV, Fixed, 95% CI
Hsiang 2020 (1)	-0.2485	0.2606	0.78 [0.47, 1.30]
Hsiang 2020 (2)	-0.4308	0.273	0.65 [0.38, 1.11]
Hsiang 2020 (3)	-0.4005	0.2667	0.67 [0.40, 1.13]
Hsiang 2020 (4)	-0.6349	0.2529	0.53 [0.32, 0.87]
Hsiang 2020 (5)	-0.734	0.2606	0.48 [0.29, 0.80]

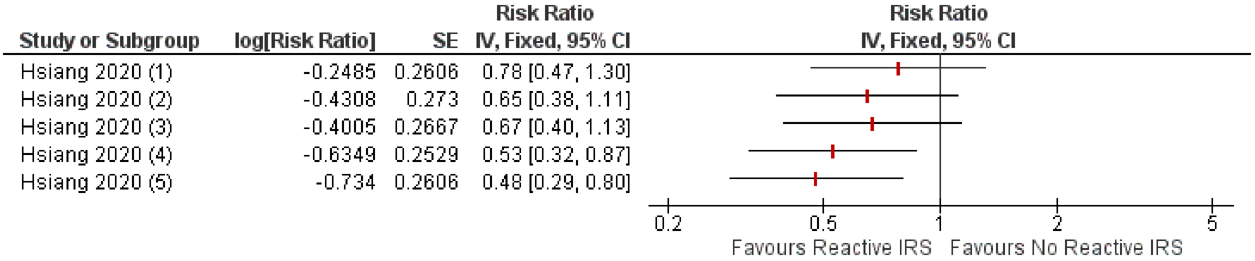

The Namibia study reported several models of the effect of reactive IRS versus no reactive IRS. The models followed stepwise inclusion of baseline incidence in 2016, the response time and coverage of reactive IRS and co-interventions. Three of the models did not indicate a statistically significant difference between the reactive IRS and no reactive IRS arms. Statistical significance was observed for model that included baseline incidence and co-interventions and the model that included baseline incidence, response time and coverage, and co-interventions. Because it was specified in the original analysis plan, the model adjusted for baseline incidence only was reported in Figure 2.

(1) Crude model;
(2) Model adjusted for baseline incidence (as reported in Figure 2);
(3) Model adjusted for baseline incidence and coverage/response time;
(4) Model adjusted for baseline incidence and co-interventions;
(5) Model adjusted for baseline incidence, coverage/response time and co-interventions

Supplemental Figure 2. Mean difference in incidence between reactive IRS and proactive, focal IRS by year in the South Africa study[18]

Study or Subgroup	Mean Difference	SE	Mean Difference IV, Fixed, 95% CI
**2.1.1 Year 1**			
Bath 2021	0.04	0.0612	0.04 [-0.08, 0.16]
**2.1.2 Year 2**			
Bath 2021	0.24	0.4337	0.24 [-0.61, 1.09]

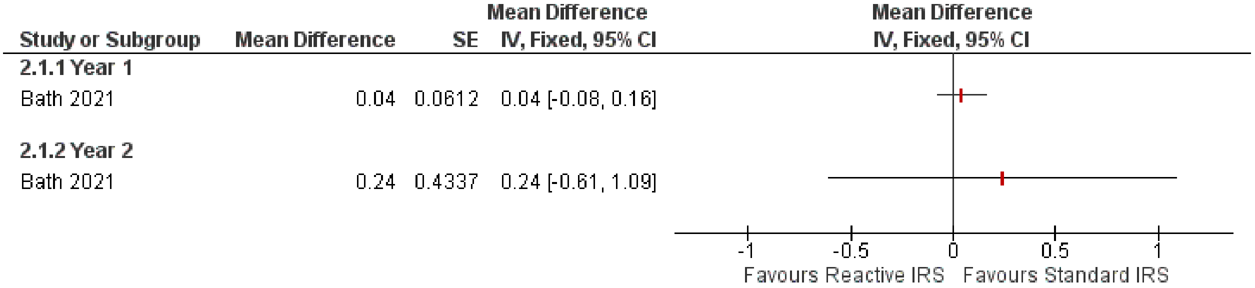

The South Africa study reported the mean difference in incidence by year and by province. In the first year, malaria incidence was low overall and the mean difference in incidence between the study arms was 0.04 cases per 1000 person-years (95% CI -0.08-0.16). The upper confidence limit did not cross the non-inferiority bound. In the second year, overall incidence of malaria was higher and the mean difference in incidence was 0.24 cases per 1000 person-years (95% CI -0.61-1.09). The 95% CI crossed the non-inferiority bound indicating that reactive IRS was non-inferior to proactive, focal IRS. However, the 90% CI did not cross the non-inferiority bound indicating that reactive IRS is non-inferior to proactive, focal IRS with 90% confidence.

Supplemental Figure 3. Mean difference in incidence between reactive IRS and proactive, focal IRS by province in South Africa[18]

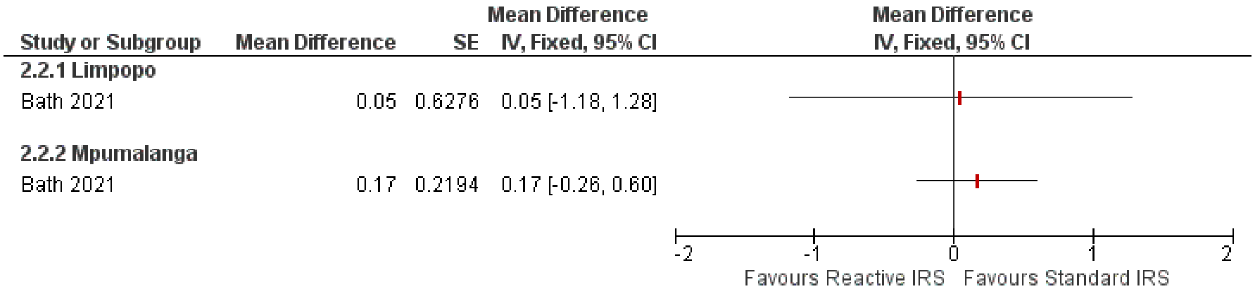

When analyzed by province, the mean difference in malaria incidence in Limpopo province was 0.05 cases per 1000 person-years (95% CI -1.18-1.28). The wide confidence intervals were due to the low number of clusters in Limpopo province (7 of 31 standard IRS clusters and 6 of 31 reactive IRS clusters were in Limpopo province). The mean difference in malaria incidence in Mpumalanga province was 0.17 cases per 1000 person-years (95% CI -0.26-0.60) and the upper CI did not cross the non-inferiority bound.

Supplemental Figure 4. Risk of bias for included studies

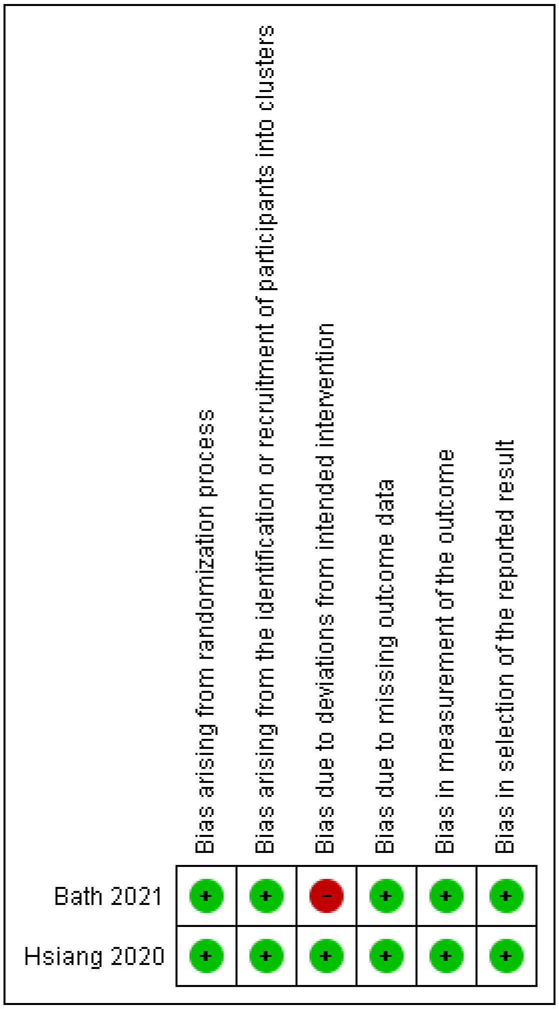

References

1. Chadee DD, Maitre Al, Tilluckdharry CC, 1992. An outbreak of Plasmodium vivax malaria in Trinidad, W.I. *Annals of Tropical Medicine and Parasitology 86*: 583-590.
2. Chanda E, et al., 2018. An investigation of the Plasmodium falciparum malaria epidemic in Kavango and Zambezi regions of Namibia in 2016. *Trans R Soc Trop Med Hyg 112*: 546-554.
3. Galappaththy GNL, Gueye CS, Sanders K, Rundi C, Vestergaard L, Cotter C, Gosling R, 2012. Reactive surveillance methods used for malaria elimination in Asia and the PacifiC: Results from a 12 country survey. *Malaria Journal 1)*: S54.
4. Galatas B, et al., 2020. A multiphase program for malaria elimination in southern Mozambique (the Magude project): A before-after study. *PLoS Medicine 17*: e1003227.
5. Gerardin J, Bever CA, Bridenbecker D, Eisele TP, Miller JM, Eckhoff PA, Wenger EA, 2017. Preventing reestablishment of malaria in recentlyeliminated areas: A modeling study of reactive case detection and adaptive response. *Am J Trop Med Hyg 97 (5 Supplement 1)*: 593-594.
6. Gueye CS, et al., 2018. Perception of malaria risk and acceptability of reactive, focal administration of presumptive treatment and indoor residual spraying, a qualitative study from the malaria elimination setting of Namibia. *Am J Trop Med Hyg 99*: 130-131.
7. Hetzel MW, Chitnis N, 2020. Reducing malaria transmission with reactive focal interventions. *Lancet 395*: 1317-1319.
8. Huda MM, Ghosh D, Alim A, Almahmud M, Olliaro PL, Matlashewski G, Kroeger A, Mondal D, 2019. Intervention packages for early visceral leishmaniasis case detection and sandfly control in Bangladesh: A comparative analysis. *Am J Trop Med Hyg 100*: 97-107.
9. Kandeel A, Haggag AA, El Fetouh MA, Naiel M, Refaey SA, Hassan AH, Ramzy RM, 2016. Control of malaria outbreak due to Plasmodium vivax in Aswan Governorate, Egypt. *East Mediterr Health J 22*: 274-9.
10. Karunasena VM, et al., 2019. The first introduced malaria case reported from Sri Lanka after elimination: implications for preventing the re-introduction of malaria in recently eliminated countries. *Malaria Journal 18*: 210.
11. Kleinschmidt I, et al., 2017. Is targeted reactive vector control a noninferior substitute for generalized indoor residual spraying in areas of very low malaria transmission-results from a cluster randomized trial. 66th Annual Meeting of the American Society of Tropical Medicine and Hygiene. Baltimore, MD: American Journal of Tropical Medicine and Hygiene, 134.
12. Larsen DA, et al., 2015. Malaria surveillance in low-transmission areas of Zambia using reactive case detection. *Malaria Journal 14*: 465.
13. London School of Hygiene and Tropical Medicine, 2015. *Targeted indoor residual spraying against malaria*. Identifier: NCT02556242. https://clinicaltrials.gov/show/NCT02556242. Accessed 6 July 2022.
14. Medzihradsky OF, et al., 2018. Study protocol for a cluster randomised controlled factorial design trial to assess the effectiveness and feasibility of reactive focal mass drug administration and vector control to reduce malaria transmission in the low endemic setting of Namibia. *BMJ Open 8*: e019294.
15. Ntuku HM, et al., 2017. Evaluating the effectiveness and feasibility of reactive targeted parasite elimination vs. reactive case detection, with and without reactive vector control, as a community level intervention in response to confirmed, passively identified malaria cases in Zambezi region, Namibia: preliminary results from a cluster randomized controlled trial. 66th Annual Meeting of the American Society of Tropical Medicine and Hygiene. Baltimore, MD: American Journal of Tropical Medicine and Hygiene, 127.

16. University of California San Francisco, 2016. *Evaluation of Reactive Focal Mass Drug Administration (rfMDA) +/- Reactive Focal Vector Control (RAVC) in Namibia*. Identifier: NCT02610400. https://ClinicalTrials.gov/show/NCT02610400. Accessed 6 July 2022.
17. Hsiang MS, et al., 2020. Effectiveness of reactive focal mass drug administration and reactive focal vector control to reduce malaria transmission in the low malaria-endemic setting of Namibia: a cluster-randomised controlled, open-label, two-by-two factorial design trial. *Lancet 395*: 1361-1373.
18. Bath D, et al., 2021. Effectiveness and cost-effectiveness of reactive, targeted indoor residual spraying for malaria control in low-transmission settings: a cluster-randomised, non-inferiority trial in South Africa. *Lancet 397*: 816-827.
